# Trypophobia, skin disease, and the visual discomfort of natural textures

**DOI:** 10.1038/s41598-024-55149-8

**Published:** 2024-02-29

**Authors:** Christopher DiMattina, R. Nathan Pipitone, Martin R. Renteria, Kriston J. Ryan

**Affiliations:** 1 Department of Psychology, Florida Gulf Coast University, Fort Myers, FL 33965-6565 USA; 2 Department of Biological Sciences, Florida Gulf Coast University, Fort Myers, FL 33965-6565 USA

**Keywords:** Neuroscience, Psychology

## Abstract

In the last decade, the behavioral sciences have described the phenomenon of trypophobia, which is the discomfort felt by some individuals when viewing images containing clusters of bumps or holes. One evolutionary hypothesis for this phenomenon is that this visual discomfort represents an adaptation which helps organisms avoid skin disease and/or ectoparasites. Even though trypophobic imagery and disease imagery are both examples of visual textures, to date there has been no comparison of the visual discomfort elicited by these two specific kinds of textures within the larger context of the visual comfort elicited by natural texture imagery more generally. In the present study, we administered the Trypophobia Questionnaire (**TQ**) and recorded the visual comfort ratings elicited by a large set of standard natural texture images, including several trypophobic and skin disease images. In two independent samples, we found that while all observers find skin diseases uncomfortable to view, only those scoring high on the TQ rated trypophobic imagery equally uncomfortable. Comparable effects were observed using both standard ANOVA procedures as well as linear mixed effects modeling. Comparing the ratings of both high-TQ and low-TQ participants to the standard textures, we find remarkably consistent rank-order preferences, with the most unpleasant textures (as rated by both groups) exhibiting qualitative similarities to trypophobic imagery. However, we also find that low-level image statistics which have been previously shown to affect visual comfort are poor predictors of the visual comfort elicited by natural textures, including trypophobic and disease imagery. Our results suggest that a full understanding of the visual comfort elicited by natural textures, including those arising from skin disease, will ultimately depend upon a better understanding of cortical areas specialized for the perception of surface and material properties, and how these visual regions interact with emotional brain areas to evoke appropriate behavioral responses, like disgust.

## Introduction

**Figure 1:**
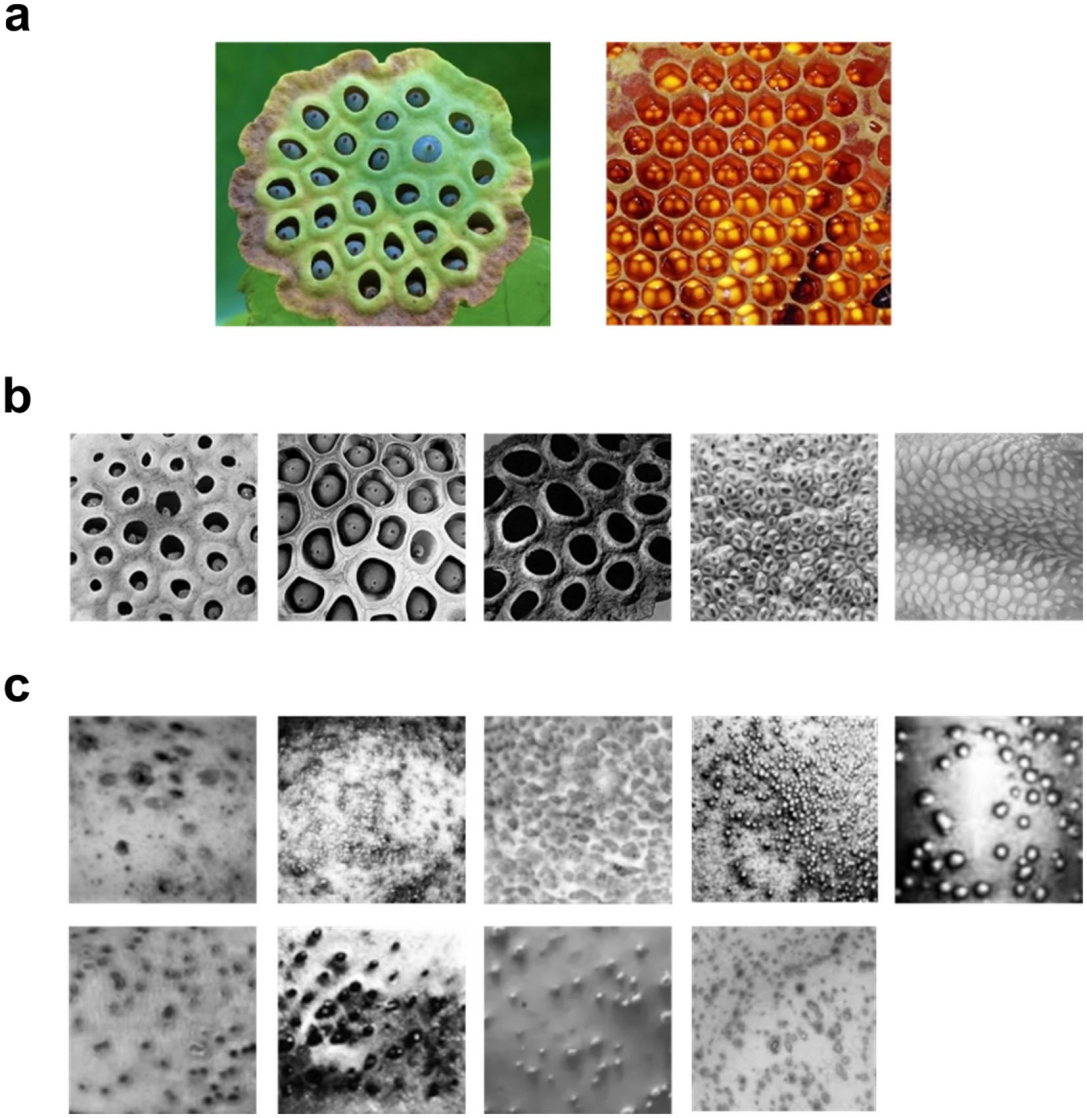
Typophobic (**TRY**) and disease (**DIS**) images used in this study. (**a**) Images used for the TQ survey. (**b**) Trypophobic images used in the experiment, used previously in Ref.^25^. (**c**) Images of the skin diseases used in the experiment. *Top row:* acne, herpes, pitted keratolysis, pustular psoriasis, smallpox-1. *Bottom row:* smallpox-2, blackheads, chicken pox-1, chicken pox-2.

Trypophobia is defined in the behavioral sciences literature as the visual discomfort experienced by some people when viewing irregular clusters of roughly circular shapes^1,2^. The aversive response elicited by trypophobic imagery is typically characterized by feelings of disgust^3^ and some examples of images which commonly elicit trypophobic reactions are shown in Fig. 1a,b. Several previous studies have shown that a sizeable minority of people (7–15%) qualify as trypophobic^1^, and furthermore many people who do not meet the formal criterion for trypophobia^2^ find trypophobic imagery uncomfortable^3–5^. Given the harmless nature of the stimuli which elicit trypophobia, its relatively high prevalence in the general population is rather mysterious, and several theories have been proposed as possible explanations^6^. Perhaps the currently best-supported theory is that trypophobia represents an over-generalization of an adaptive disgust response to stimuli which visually resemble skin diseases^3,6–8^, thereby functioning as a component of the behavioral immune system^9–13^.

As we can see from the images in Fig. 1, both trypophobic (Fig. 1a,b) and skin-disease (Fig. 1c) images are examples of visual textures, exhibiting quasi-periodic spatial repetition of smaller elements sometimes referred to as “textels” or “micro-patterns”^14–17^. Just as information about objects (“things”) is primarily conveyed by edges and shape, information about materials (“stuff”) is primarily conveyed by visual texture^18–20^. Unlike visual objects, visual textures are defined statistically: As one samples from different sub-regions of a larger texture image, one gets roughly similar images having a similar appearance and statistical properties^21^. By contrast, if one samples from sub-regions of an image of an object (e.g., a face) one gets very different images (ear, eye, etc.…). Despite the fact that trypophobic stimuli are natural textures, they have not been well-studied in the context of texture perception and have not been explicitly tested in the literature on visual discomfort elicited by textures^22^, although there have been efforts to explain trypophobia in terms of lower-level image statistics^1,4,23,24^. The literature on trypophobia has generally used non-texture control stimuli, for instance, images containing circular objects and/or single holes^2,25,26^, or very limited sets of control textures with some visual characteristics (e.g., holes) which match trypophobic imagery^3^. In fact, to the best of our knowledge, a systematic comparison of the visual comfort elicited by trypophobic stimuli and a broad set of representative natural texture stimuli has not yet been performed in the behavioral sciences literature.

Furthermore, no efforts to date have been made to understand the extent to which trypophobic and non-trypophobic individuals exhibit preference similarities or differences for natural textures, and only one study that we are aware of has directly compared responses of these two populations to skin disease and trypophobic images^3^. Finally, although some studies have related statistical image properties to texture aesthetics^22^, studies focused on visual comfort have typically used artificial textures^27–32^, rather than natural textures.

The goal of the present study is to help close this gap between the texture perception and trypophobia literatures. Utilizing the Trypophobia Questionnaire (**TQ**)^2^, we measured levels of trypophobia in a sample of participants and define high-trypophobia (**hi-TQ**) and low-trypophobia (**lo-TQ**) populations. We measured the visual comfort elicited by a set of 56 representative natural textures from the Brodatz (**BDZ**) database used in previous psychophysical studies^33,34^, together with the visual comfort elicited by 5 trypophobic textures^25^ and 9 disease (**DIS**) textures. We show that while **lo-TQ** and **hi-TQ** people find images of disease similarly unpleasant, only **hi-TQ** individuals find **TRY** images as unpleasant as those of disease. Using linear mixed effects modeling, which treats TQ scores quantitatively and estimates cross random effects of different participants and image types, yielded similar results. Analyzing the data on an image-by-image basis, we rank the Brodatz textures from the least to the most comfortable, for both **hi-TQ** and **lo-TQ** populations. We find a broad qualitative agreement between these populations that the most comfortable images tend to be low-density, highly regular, and often have a dominant orientation, whereas the most discomforting images tend to be high density, highly irregular, and often contain circular clusters, qualitatively similar to trypophobic imagery. We identified several Brodatz textures which are significantly less comfortable for the **hi-TQ** participants than the **lo-TQ** participants, and these resemble the **TRY** stimuli in containing clusters of holes or bumps. But across the corpus of texture stimuli, there was a strong correlation between ratings from both populations, suggesting that even **lo-TQ** individuals find stimuli which share similar visual characteristics with **TRY** stimuli less comforting. Furthermore, we see strong agreement between **hi-TQ** and **lo-TQ** populations in their visual comfort ratings for skin disease stimuli, despite their vastly different responses to **TRY** stimuli.

Finally, we considered the relationship between visual comfort and simple statistics measured from the Fourier amplitude spectra of our natural visual textures. Previous literature has suggested that deviations from the $1/f$ (orientation-averaged) amplitude spectrum characteristic of most natural images^35,36^ can lead to visual discomfort, particularly in cases where there is an over-representation of mid-range (~ 3 cycles/degree of visual angle) spatial frequencies^23,24,32,37,38^. More recent work with noise images^29,30^ has also demonstrated some preference for images which are narrowband in their (spatial frequency averaged) orientation spectrum (*orientation anisotropy*), in other words they contain a single dominant orientation (for instance, a field of wheat). Since trypophobic stimuli and images of skin disease are broadband in their (spatial frequency averaged) orientation spectrum (*isotropic*), and their amplitude spectrum over-represents mid-range spatial frequencies, one reasonable hypothesis is that these two textures represent a “perfect storm” of cues that normally evoke visual discomfort in images more generally.

In our analysis of basic image statistics, we find that many of the characteristics which have been proposed to explain visual discomfort do not differ significantly between trypophobic images and generic textures. Most notably, we find that much like trypophobic images and images of skin disease, texture images in general tend to over-represent mid-range spatial frequencies relative to $1/f$. Measuring these statistical characteristics from individual images shows that they are relatively poor predictors of visual comfort ratings for texture images, and greatly under-predict the visual discomfort elicited by trypophobic imagery or by images of disease. We suggest that since natural textures can vary along many perceptual dimensions which are not captured by simple statistics, a more careful characterization of texture statistics is needed to develop useful predictive models of visual comfort^22,39^. We also suggest that the brain mechanisms utilized for general texture/materials perception possess the ability to recognize potentially harmful materials. Therefore, while cortical hypermetabolism in early visual areas may be able to explain visual discomfort for images more generally (see Ref.^23^ for effects when viewing trypophobic imagery), for behaviorally relevant images like skin disease we suggest that more specialized mechanisms in higher visual areas having interconnections with emotional brain regions are responsible for evoking appropriate behavioral responses, like disgust.

## Methods

### Image sets

To compare the visual comfort elicited by natural textures, trypophobic stimuli, and disease stimuli, we made use of multiple resources. Our natural texture set was comprised of 56 representative natural textures from the Brodatz (**BDZ**) database used in previous psychophysical research^34^. These 56 images were sampled from the full version of the 112 image original Brodatz dataset obtained from a computer vision laboratory website: https://multibandtexture.recherche.usherbrooke.ca/original_brodatz.html. Since the images in this database (and many other online databases) were contrast-reversed from those in the original album^40^, we also tested these same images with inverted contrast polarity in a second study. In this paper, we will refer to these two versions of our study with different versions of the Brodatz images as the *pilot* and *main*, respectively, and in many analyses the results will be pooled. Our set of trypophobia-inducing stimuli (**TRY**) was a set of 5 textures found in previous research to cause the greatest trypophobic visual discomfort, illustrated in Fig. 1b^25^. Finally, 9 disease (**DIS**) textures were obtained from the internet, representing a variety of skin conditions, including smallpox, pitted keratolysis, and cystic acne (Fig. 1c). All test images were presented in grayscale and resized to 256 × 256 resolution and are available for download at: https://www.fgcu.edu/faculty/cdimattina/. The images used for administration of the TQ (Fig. 1a) were presented in color, following previous research^2,5,6,25^.

### Participants

All participants were students taking psychology courses at Florida Gulf Coast University (FGCU). As compensation, students received course credit as specified in their syllabi. Responses from all participants who completed the Trypophobia Questionaire (**TQ**, see below for details) and responded to all questions were considered valid and included in the analysis. Each participant signed a consent form by pressing a button at the beginning of the survey. All procedures were approved beforehand by the FGCU IRB (**Protocol 2022-12**), in accordance with the Declaration of Helsinki. Two separate samples were obtained during recruitment for this study. Details of these samples are discussed below.

### Description of TQ

The Trypophobia Questionnaire (**TQ**) was created in a prior study to better assess the degree to which an individual may experience disgust or discomfort when introduced to trypophobic images^2^. This measure has been utilized previously in multiple studies^6,25^, and has been independently validated in previous research^5^. The TQ was administered using Qualtrics. Participants looked at the two images shown in Fig. 1a while answering a set of 17 questions on a 1–5 scale indicating the feelings elicited by the images, with 1 indicating no discomfort/aversion and 5 a high degree of discomfort/aversion. The scores for all questions were tallied to obtain a final score in the range from 17 to 85. According to the group which defined this survey, a score greater than 31 is the cut-off to be considered trypophobic^2^.

### Qualtrics survey

The self-report survey utilizes Qualtrics software (www.qualtrics.com), and observers completed the survey online using their personal electronic devices (phones, tablets, laptops or PCs). At the start of the survey, participants read an informed consent form detailing the experiment’s purpose and its voluntary nature. Since it was an online survey, participants signed the informed consent form by pressing a button. If a participant consented to the survey, they provided basic non-identifiable demographic information and then took the Trypophobia Questionnaire (**TQ**). The main part of our survey consisted of 70 questions where participants rated the visual comfort of images taken from our image sets. These include the 56 Brodatz (**BDZ**), 5 trypophobic (**TRY**), and 9 disease (**DIS**) images described above. The rating scale measured viewing comfort on an 11-point Likert scale ranging from -5 to 5, with -5 being “very uncomfortable”, 0 being “neutral”, and 5 being “very comfortable.”

### Data cleaning

Data was cleaned by eliminating any duplicate responses so to the best of our knowledge there was only one response per participant. Any participant who did not complete all 17 questions on the Trypophobia Questionnaire (**TQ**) was also eliminated, as was any participant who did not rate any images in one of the three categories (**BDZ**, **DIS**, **TRY**). When pooling data from the pilot and main studies, we found that a small number of individuals were participants in both studies (which were conducted in different academic years), so we removed these duplicates as well.

### Data analyses

While analyzing data, defining high-TQ/low-TQ populations was a factor of interest. To define populations of high-TQ (**hi-TQ**) and low-TQ (**lo-TQ**) individuals for comparison, we utilized *median split grouping* in which the median of the continuous variable is found so that any value below it would be considered “low” and any value higher than it would be considered “high.” Using this categorical definition of high-TQ and low-TQ populations, we then performed a mixed-effects 2-way ANOVA, with between-subjects factor of TQ score (2 levels: **lo-TQ**, **hi-TQ**) and within-subjects factor of image category (3 levels: **BDZ**, **TRY**, **DIS**). The ANOVA analysis was performed using JASP^®^ (https://jasp-stats.org) for both the original sample, main sample, and pooled sample. Similar results were obtained when we performed a *definitional split grouping* where we defined the **lo-TQ** and **hi-TQ** groups by comparing the participants whose score was greater than the cut-off (> 31 which defines having trypophobia) to an equal number of participants with the lowest scores.

Although the impact that trypophobia has can be easily seen when splitting participants into **lo-TQ**/**hi-TQ** groups, TQ scores do vary on a continuum. Therefore, we also implemented a linear mixed-effects model using the statistical software Jamovi 2.4.5 (https://www.jamovi.org). Using a mixed model approach allowed us to investigate the impact of TQ scores as a quantitative variable, and since participants provided ratings of multiple images from each image category, we could specify a two-level random intercept model which estimates cross random effects of participant and image type while calculating the impact of the fixed effects (predictor variables) used in the study. Mathematically, the model was given by the expression

1 $${{Comfort }}\sim { 1 } + {{ Image\, Type }} + {{ TQ \,Score }} + {{ Image\, Type}}:{{TQ\, Score}} + \, \left( {{1}|{{Item\, ID}}} \right) \, + \, \left( {{1}|{{Subject\, ID}}} \right),$$

where *(1|Item ID)* and *(1|Subject ID)* represent item and subject-dependent intercepts and *Image Type:TQ* score represents an interaction term allowing TQ score to have different effects for different image categories.

### Image analyses

We also measured basic image statistics which have been previously associated with visual discomfort like mean luminance, contrast, and over-representation of mid-range spatial frequencies relative to the $1/f$ amplitude spectra characteristic of most natural images^35,36^. Mean luminance was characterized for each image by simply averaging the pixel intensities. RMS contrast was obtained for each image as the standard deviation of pixel intensities divided by the mean luminance. Somewhat complicating our measurement of over-representation of mid-range spatial frequencies (**mid-SF**) is that viewing distance and image size were not controlled since the survey was administered online (but if viewing on a smartphone, most participants would be using similar distances). Therefore, in a strict sense we can only specify the mid-SF range in terms of cycles/image (**cpi**) rather than cycles/degree (**cpd**) of visual angle. However, assuming an average image size on the observer’s retina of approximately 4 deg. of visual angle, we measured excess energy relative to $1/f$, making use of three definitions of mid-range spatial frequencies. The first definition of the mid-SF range was 2–9 cpd (**2to9**), taken from^24^. The second definition was 3 cpd +/− 1 octave (**1oct3**), or 1.5–6 cpd^37^. Finally, the third definition was 3 cpd +/− 2 octaves (**2oct3**), or 0.75–12 cpd^25^. For our 256-pixel images, these definitions correspond to 8–36, 6–24**,** and 3–48 cycles/image, respectively.

More recent work using noise textures has also suggested that textures which are narrowband in orientation are more comfortable to view than those which are more broadband in orientation^29,30^. Such textures will typically be comprised of a single dominant orientation (for instance a field of wheat), and we refer to this non-uniformity of orientation content as *orientation anisotropy*. By contrast, most natural images without a single dominant orientation will tend to have an approximately uniform distribution of orientations (with slight over-representation of cardinal orientations), and broadband noise will have a complete uniform distribution of orientation, a situation referred to as *orientation isotropy*. To quantify the orientation anisotropy in our set of textures, we took the 2-D Fourier transform and averaged the amplitude spectrum over spatial frequency for $n=25$ equal intervals of orientations from $\left[-\pi ,\pi \right]$. This gave us for each image a probability distribution $p\left(\theta \right)$ of energy at each orientation. We then computed the symmetrized Kullbeck-Lieber (KL) divergence ${D}_{\parallel }\left(p,q\right)$ between this distribution of oriented energy and the distribution $q\left(\theta \right)$ of oriented energy for broadband noise. The symmetrized KL distance is defined by

2 $${D}_{\parallel }\left(p,q\right)=\frac{{D}_{KL}\left(p,q\right)+{D}_{KL}\left(q,p\right)}{2} ,$$

where

3 $${D}_{KL}\left(p,q\right)= {\int }_{-\pi }^{\pi }p\left(\theta \right){\text{ln}}\frac{p\left(\theta \right)}{q\left(\theta \right)}d\theta .$$

The larger the ${D}_{\parallel }\left(p,q\right)$, the greater the orientation anisotropy, with ${D}_{\parallel }\left(p,q\right)=0$ representing no anisotropy (perfect isotropy). The value of this measure will generally be largest for textures with a single, dominant orientation, and will be the smallest for textures without a single dominant orientation. This is of interest for the current study because trypophobic imagery is characterized not only by over-representation of mid-SF energy, but also being broadband in its orientation content since it is comprised of circular regions and bumps. Therefore, a reasonable hypothesis to consider is whether the broadband orientation spectra of **TRY** imagery contribute to the visual discomfort elicited by these images.

## Results

### TQ survey results and group definitions

**Figure 2:**
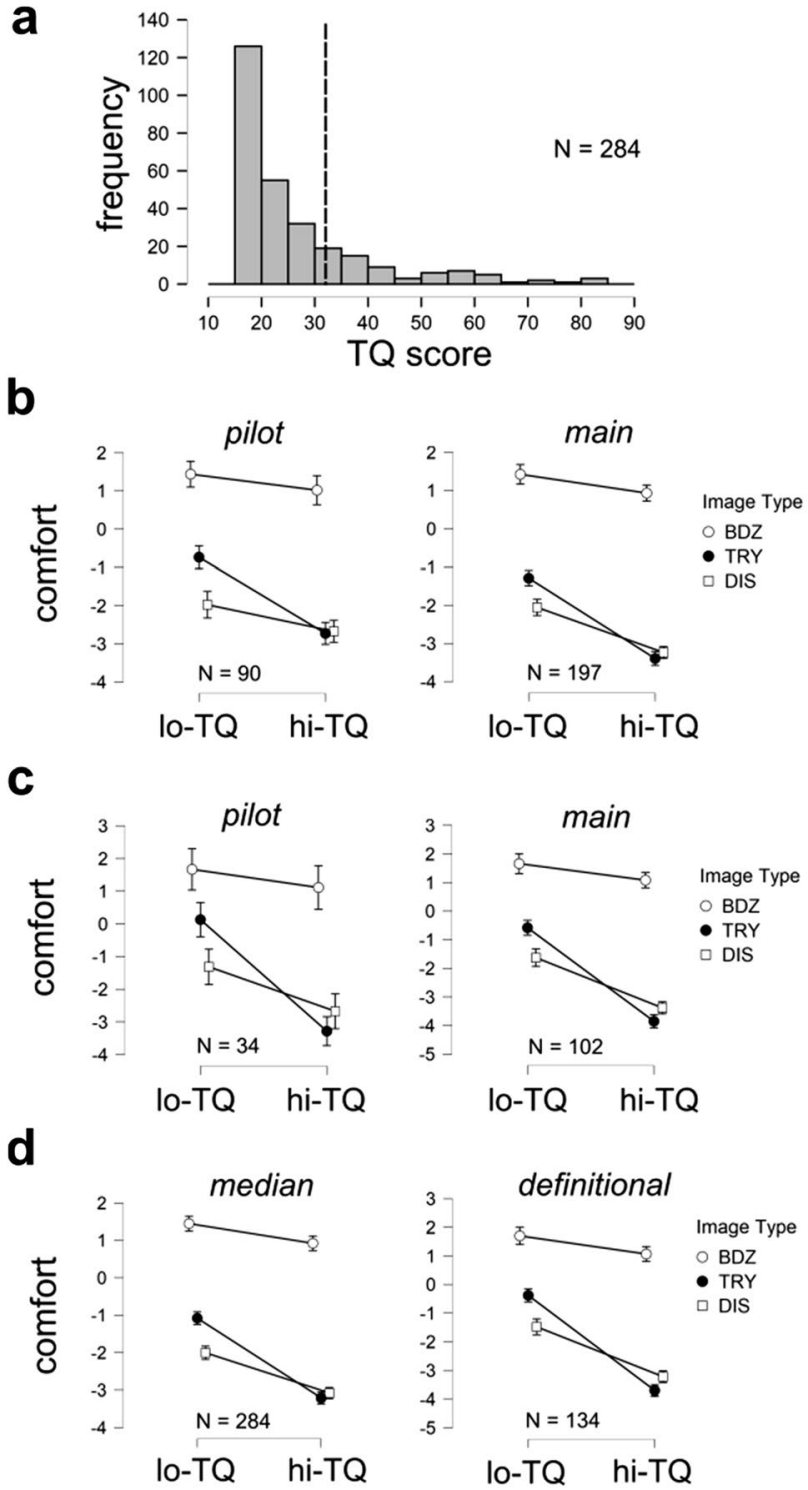
Distribution of TQ scores and mean visual comfort ratings. (**a**) Distribution of scores on the Trypophobia Questionnaire or TQ^2^. Dashed line indicates the cut-off TQ score which determines definitional trypophobia. (**b**) Mean visual comfort ratings for different categories of images (Brodatz, Disease, Trypophobic) images for **lo-TQ** and **hi-TQ** populations for median split grouping. *Left:* Pilot survey, *Right:* Main survey. Error bars indicate 95% confidence intervals. (**c**) same as (**b**) but for definitional split grouping. (**d**) Same as (**b**) and (**c**) but pooled across the pilot and main surveys. *Left:* Median split. *Right:* Definitional split.

Figure 2a shows the distribution of TQ scores obtained from the N = 284 unique participants pooled across the pilot study (N = 90) and main study (N = 197). TQ scores in our sample varied from 17–85, with a score greater than 31 being the cutoff to be considered trypophobic^2^. The median participant had a TQ of 21 and 67 of the 284 participants had a TQ score greater than 31 (23.6%), broadly consistent with the proportions reported (7%-17%) in previous studies^1,5,25^.

### Visual comfort ratings by group and image category

#### General observations

To test whether **hi-TQ** and **lo-TQ** participants gave different ratings to different categories of images, we compared the mean comfort ratings for each of the three image categories (**BDZ, DIS, TRY**) between the two groups of participants. Defining the two populations using a simple median split, we see from Fig. 2b that for both the pilot and main studies that on average the Brodatz textures are rated by all groups as being as somewhat more comfortable than the neutral mid-point of the scale (0), whereas all groups rate both the disease and trypophobic images as being less comfortable on average than the neutral mid-point. Also, those who score higher on the TQ exhibit a greater discomfort when viewing trypophobic images, qualitatively replicating previous results using these same images^25^. Similar results were obtained when the **hi-TQ** and **lo-TQ** groups were specified using the definitional split (Fig. 2c).

Comparing the mean comfort ratings for the Brodatz textures in the pilot (contrast reversed) and main (original contrast) surveys using a two independent sample *t*-test failed to reveal a significant difference (*t*(282) = 0.323, *p* = 0.747). This demonstrates that the contrast-reversal operation has no effect on average visual comfort for the Brodatz textures, and therefore we pool data across surveys for ANOVA analyses presented below.

#### ANOVA results

We performed a 2 × 3 mixed-model ANOVA with the group (**lo-TQ**, **hi-TQ**) as the between-subjects variable (defining groups by a median split or definitional split), and the image category as the within-subjects variable. For the median split grouping, we observed statistically significant (α = 0.05) effects of group (*F*_1,282_ = 66.243, *p* < 0.001, η^2^ = 0.068, η_p_^2^ = 0.190) image type, (*F*_2,564_ = 1077.678, *p* < 0.001, η^2^ = 0.493, η_p_^2^ = 0.793) and interaction (*F*_2,564_ = 42.672, *p* < 0.001, η^2^ = 0.020, η_p_^2^ = 0.131). Pairwise post-hoc comparisons between image categories (pooling across groups) yielded significant differences between all image categories (**BDZ**-**TRY**: mean difference = 3.333, *t* = 37.765, *p* < 0.001; **BDZ**-**DIS**: mean difference = 3.730, *t* = 42.268, *p* < 0.001; **TRY**-**DIS**: mean difference = 0.397, *t* = 4.503, *p* < 0.001). All pairwise post-hoc comparisons of the interaction between group and image type are shown in Supplementary Table 1. We find that for all image categories, **hi-TQ** participants rate the images as being less comfortable to view than **lo-TQ** participants (**BDZ**: mean difference = 0.527, *t* = 2.87, *p* = 0.009; **TRY**: mean difference = 2.131, *t* = 11.601, *p* < 0.001; **DIS**: mean difference = 1.074, *t* = 5.844, *p* < 0.001).

Similar results were obtained for the definitional split grouping, also observing effects of group (*F*_1,132_ = 54.032, *p* < 0.001, η^2^ = 0.130, η_p_^2^ = 0.290), image type (*F*_2,264_ = 557.862, *p* < 0.001, η^2^ = 0.412, η_p_^2^ = 0.809), and interaction (*F*_2,264_ = 59.335, *p* < 0.001, η^2^ = 0.044, η_p_^2^ = 0.310). As before, significant differences were found between all image types (**BDZ**-**TRY**: mean difference = 3.423, *t* = 27.595, *p* < 0.001; **BDZ**-**DIS**: mean difference = 3.734, *t* = 30.097, *p* < 0.001; **TRY**-**DIS**: mean difference = 0.310, *t* = 2.503, *p* = 0.013). All pairwise post-hoc comparisons of the interaction between group and image type are shown in Supplementary Table 2. We find a significant difference between **lo-TQ**, **hi-TQ** groups for all image categories (**BDZ**: mean difference = 0.633, *t* = 2.143, *p* = 0.033; **TRY**: mean difference = 3.322, *t* = 11.245, *p* < 0.001; **DIS**: mean difference = 1.743, *t* = 5.899,* p* < 0.001).

#### Linear mixed effects model analysis

Although dividing the participants into two groups (**lo**/**hi**-**TQ**) is a simple and intuitive methodology to explore the relationship between TQ scores and visual comfort ratings, as we see from the histogram in Fig. 2a, trypophobia is a matter of degree rather than a matter of kind. Therefore, we also conducted a linear mixed-effects model analysis (see “Methods” for details) to investigate the impact of the continuous predictor variable (TQ score) on the outcome variable (viewing comfort), with random effects of participant and image type.

**Figure 3:**
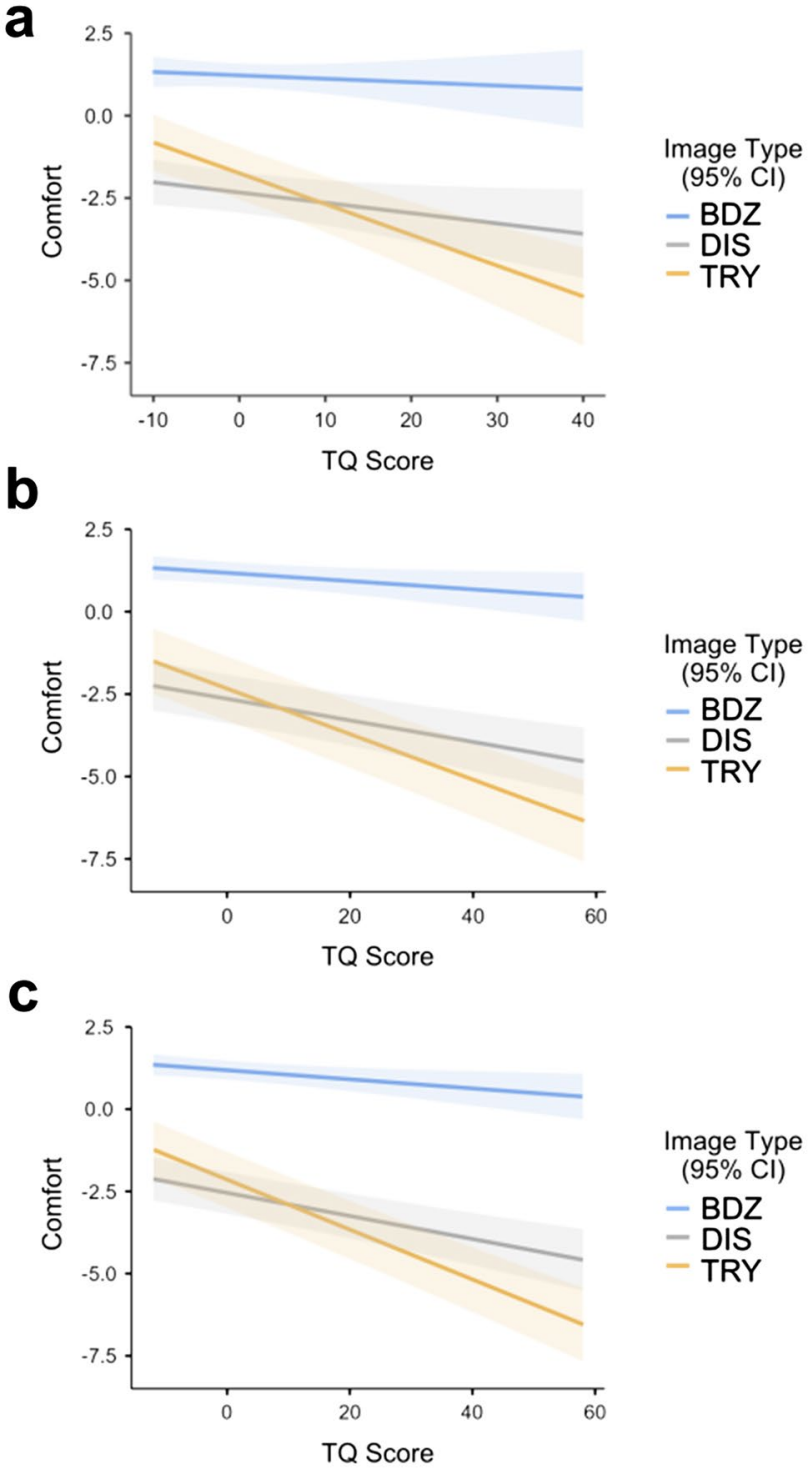
Linear mixed effects regression plots of viewing comfort as a function of TQ score for all three image categories. (**a**) Pilot study. (**b**) Main study. (**c**) Pooled.

In the pilot study (N = 90 participants), we observed significant effects of image category (*F*_2,67_ = 93.00, *p* < 0.001), TQ scores (*F*_1,93.6_ = 9.76, *p* = 0.002), and the interaction of image category and TQ scores (*F*_2,6135_ = 58.29, *p* < 0.001) on viewing comfort. We observed statistically significant negative slopes in the plots of TQ scores versus viewing comfort (Fig. 3a) for the **DIS** (*t*(113.8) = − 2.07, *p* = 0.041) and **TRY** (*t*(140.4) = − 5.858 *p* < 0.001) imagery, but not the **BDZ** images (*t*(88.9) = − 0.724, *p* = 0.471). See Supplementary Table 3 for full results and Fig. 3a for a graphical depiction of the data.

Similar results were obtained from the main study (N = 197 participants), with significant effect of image category (*F*_2,67_ = 66.8, *p* < 0.001), TQ scores (*F*_1,213.3_ = 42.1, *p* < 0.001), and their interaction (*F*_2,13514_ = 121.9, *p* < 0.001) on viewing comfort. Significant effects of TQ scores on viewing comfort (negative slopes, see Fig. 3b) were observed for all image categories (**BDZ**: *t*(198) = − 2.17, *p* = 0.031, **TRY**:* t*(372) = − 10.32, *p* < 0.001, **DIS**:* t*(281) = − 5.20, *p* < 0.001). See Supplementary Table 3 for full results and Fig. 3b for a graphical depiction of the data.

Pooling data across the pilot and main (N = 284) yielded similarly significant effects of image category (*F*_2,67_ = 82.7, *p* < 0.001), TQ score (*F*_1,306.4_ = 54.4, *p* < 0.001) and interaction (*F*_2,19513_ = 169.9, *p* < 0.001), with significant negative slopes (Fig. 3c) for all image categories (**BDZ**: *t*(286) = − 2.49, *p* = 0.013, **TRY**: *t*(516) = − 11.81 *p* < 0.001, **DIS**: *t*(395) = − 5.84, *p* < 0.001). See Supplementary Table 3 for full results and Fig. 3c for a graphical depiction of the data.

### Analysis of individual images

In both the pilot and main study, we find strong consistency between **hi-TQ** and **lo-TQ** populations in their average ratings of the Brodatz textures, which are on average ranked as being similarly comfortable to view by both groups (Fig. 2b). However, even though the two populations rate the Brodatz textures similarly, they may not rank them similarly on an image-by-image basis. That is, it is possible for the two populations to have different visual comfort ratings for different images, even if the overall average rating for a particular category (**BDZ**, **DIS**) is approximately the same. Therefore, for both definitions of **hi-TQ** and **lo-TQ** groups (median/definitional split), for both the pilot and main, we analyzed the visual comfort elicited by individual Brodatz (**BDZ**) and Disease (**DIS**) textures for all participants (**ALL**), **hi-TQ** participants, and **lo-TQ** participants to investigate any rank-order differences among the groups.

**Figure 4:**
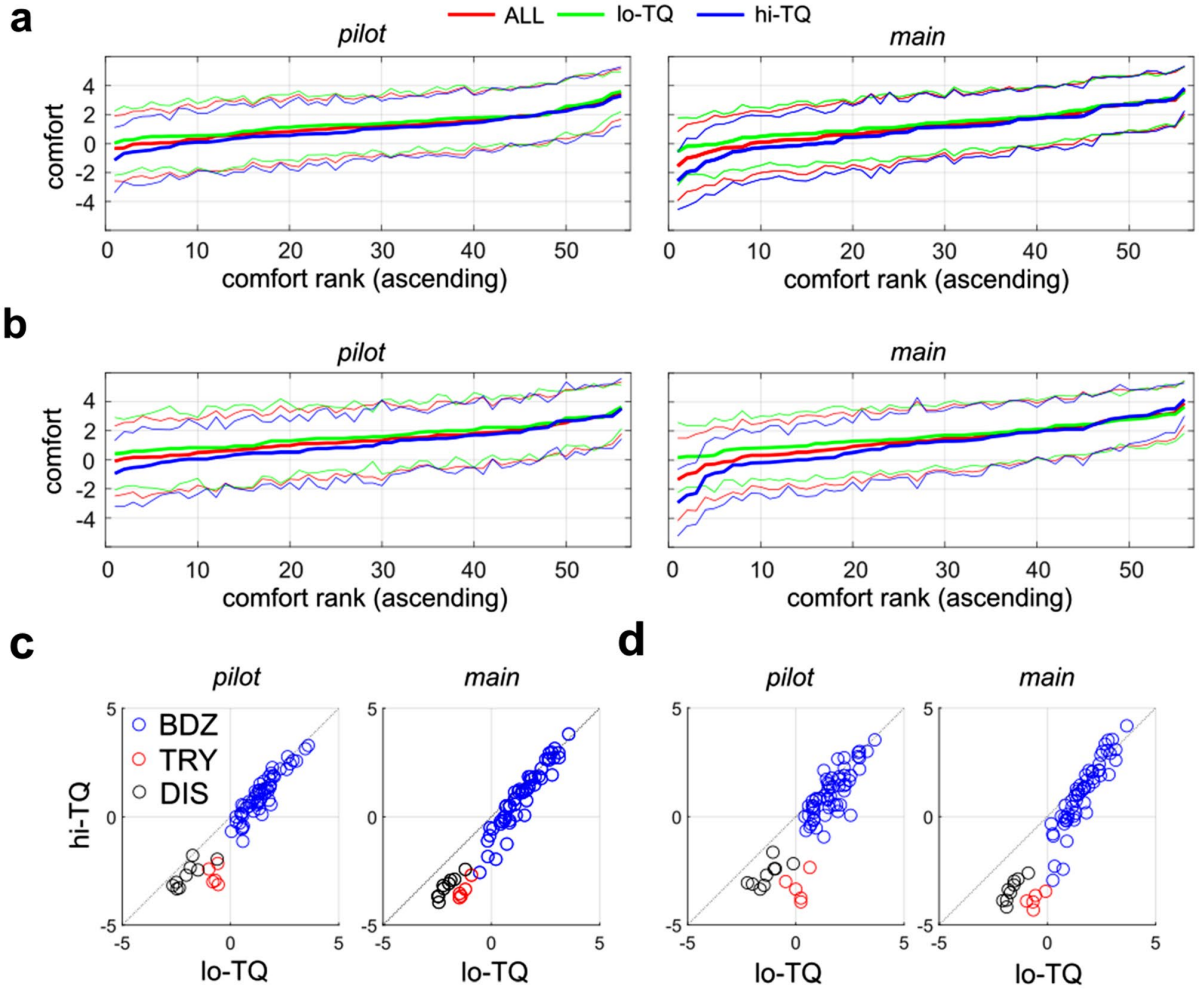
Mean visual comfort rating of Brodatz (**BDZ**) textures for different populations (**ALL**, **lo-TQ**, **hi-TQ**), for two definitions of the **lo-TQ** and **hi-TQ** populations. (**a**) Median split grouping. (**b**) Definitional split grouping. (**c**) Relationship between mean visual comfort scores for images from all three categories for **hi-TQ** and **lo-TQ** populations for median split grouping. (**d**) Same as (**c**) but for definitional split.

**Figure 5:**
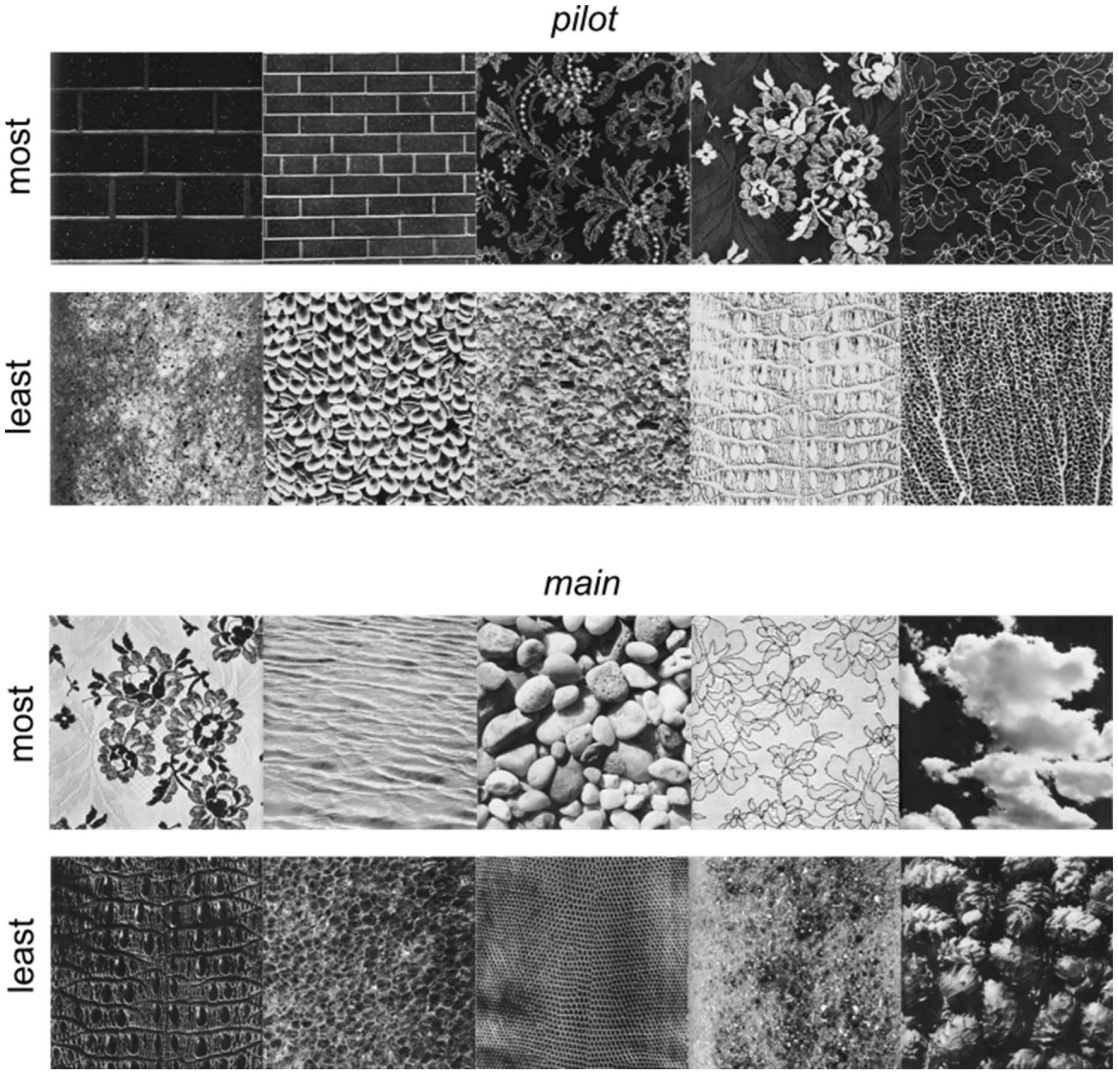
Five most comfortable and least comfortable Brodatz (**BDZ**) textures for all (**ALL**) participants. *Top:* Pilot study. *Bottom:* Main study.

**Figure 6:**
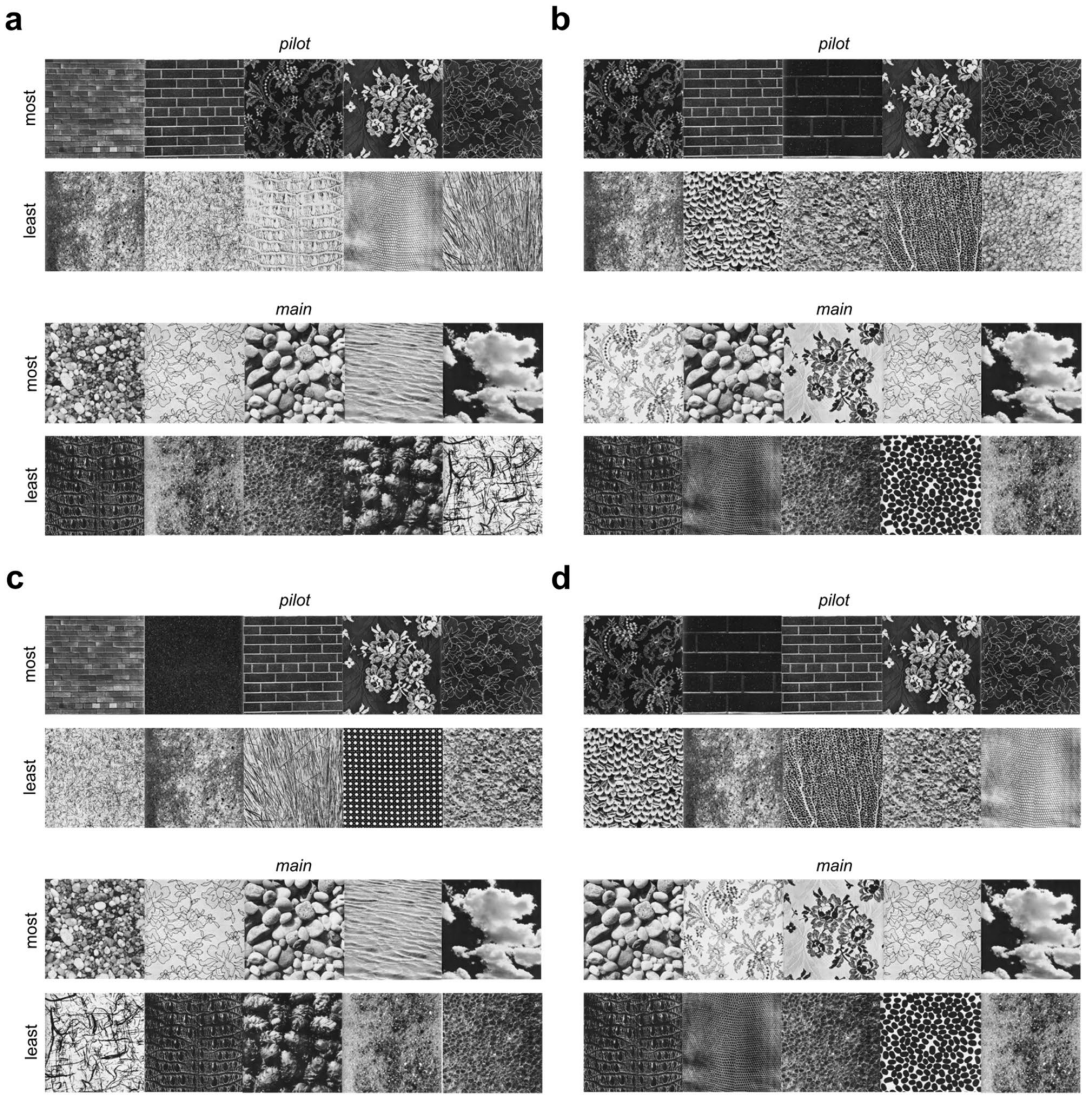
Most comfortable and least comfortable images for different populations (**lo-TQ**, **hi-TQ**) for different population groupings (median split, definitional split). (**a**) **lo-TQ** population, median split grouping. (**b**) **hi-TQ** population, median split grouping. (**c**) **lo-TQ** population, definitional split grouping. (**d**) **hi-TQ** population, definitional split grouping.

Figure 4a,b shows the **BDZ** comfort ratings for each population (mean +/− std. dev.), as a function of comfort rank (least comfortable to most comfortable) for that population (red: **ALL**, green: **lo-TQ**, blue: **hi-TQ**), for both population definitions (median split: Fig. 4a, definitional split: Fig. 4b), for both the pilot and main studies. Note that the numbers on the horizontal axis indicate ranks, with the image corresponding to that rank depending on the specific population. We see substantial overlap in the comfort ratings for the most and least comfortable images by each population, although the **hi-TQ** populations rate their most uncomfortable textures as being less comfortable on average than the l**o-TQ** participants. Figure 5 shows the 5 most comfortable images and 5 least comfortable in the Brodatz database for **ALL** participants, for both the pilot (top) and main (bottom) studies. We see that in general, the most comfortable Brodatz images are floral patterns, regular patterns like brick walls, and natural images like water, pebbles, and clouds. Interestingly, the least comfortable Brodatz images have some general qualitative similarity to trypophobic (**TRY**) imagery and disease (**DIS**) imagery (Fig. 1), being irregular, non-directional, complex, and often containing clusters of holes or bumps. Figure 6 is the same as Fig. 5, but broken down by TQ level, for two different groupings (Fig. 6a: **lo-TQ**, median split, **6b**: **hi-TQ**, median split, **6c**: **lo-TQ**, definitional split, **6d**: **hi-TQ**, definitional split). We observe a remarkable consistency between the **hi-TQ** and **lo-TQ** groups in the Brodatz textures they find to be the most and least comfortable, with both groups finding floral patterns, brick walls, and clouds to be pleasant, and Brodatz images which resemble trypophobic imagery unpleasant, consistent with our observations in Fig. 2.

To quantify the broad agreement in preference for the **BDZ** textures between **hi-TQ** and **lo-TQ** populations, we made scatterplots of the mean ratings of each of the images by both the **hi-TQ** and **lo-TQ** populations for both groupings (Fig. 4c: median split, Fig. 4d: definitional split). For the **BDZ** textures (blue symbols), we find in all cases a highly significant rank-order correlation (Spearman’s ρ) between rankings by **lo-TQ** and **hi-TQ** participants (median split, pilot: ρ = 0.907, *p* < 0.001, main: ρ = 0.964, *p* < 0.001; definitional split, pilot: ρ = 0.767, *p* < 0.001, main: ρ = 0.950, *p* < 0.001). A similar broad agreement was found for the **DIS** images (Fig. 4c,d: black symbols) for both groupings and both experiments (median split, pilot: ρ = 0.783, *p* = 0.017, main: ρ = 0.964, *p* < 0.001; definitional split, pilot: ρ = 0.745,* p* = 0.026, main: ρ = 0.954, *p* < 0.001). This suggests that both populations also agree on which disease images are most uncomfortable.

By contrast, we do not observe any significant correlations between the rankings of **hi-TQ** and **lo-TQ** populations (red symbols) for the **TRY** images (median split, pilot: ρ = − 0.300, *p* = 0.683, main: ρ = 0.900, *p* = 0.0803; definitional split, pilot: ρ = 0.051,* p* = 1.0, main: ρ = 0.600, *p* = 0.350).

**Figure 7:**
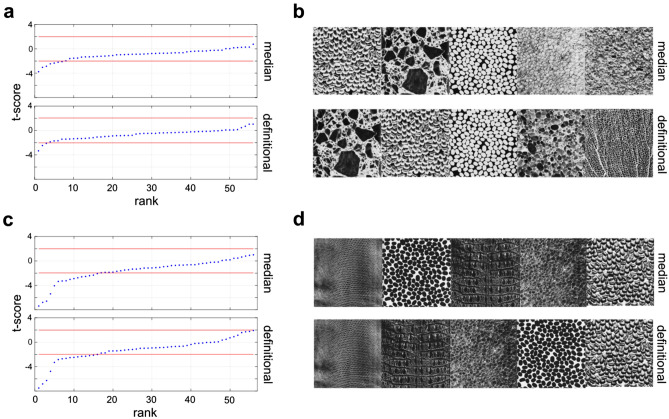
Brodatz images which best distinguish **hi-TQ** and **lo-TQ** populations. (**a**) t-scores for comparisons between **hi-TQ** and **lo-TQ** populations (sorted in ascending order) for the pilot study. Dashed lines show cutoff for statistical significance (two-tailed test, α = 0.05) *Top*: median split, *Bottom:* definitional split. (**b**) Five images which best differentiate **hi-TQ** and **lo-TQ** populations, for both splits. (**c**) Same as (**a**) but for the main study. (**d**) Same as (**b**) but for the main study.

Given that the **lo-TQ** and **hi-TQ** populations show vastly different responses to the **TRY** images (see the red symbols in Fig. 4c,d), we might posit that the Brodatz textures which would best differentiate the **hi-TQ** and **lo-TQ** groups would be those that most closely visually resemble **TRY** imagery (Fig. 1a,b). That is, such images should have clusters of holes or bumps. Performing an independent samples *t*-test for each image to compare the average comfort ratings yielded the *t*-scores shown in Fig. 7a (pilot) and Fig. 7c (main). We see that the only differences which reach statistical significance (dashed black lines) are those for which the **hi-TQ** population had significantly lower comfort ratings than the **lo-TQ** population (negative *t-*score). In no case did the images rated as being more comfortable by the **lo-TQ** population reach statistical significance. The five images best differentiating the two populations are shown for both the pilot (Fig. 7b) and main (Fig. 7d) studies. We see that these images do indeed visually resemble trypophobic imagery, being comprised of clusters of bumps or holes (see Fig. 1a,b).

### Image statistics and visual comfort

#### Basic image statistics

We measured from each image some basic statistics which have been suggested previously to affect visual comfort, including mean luminance (**lum**), RMS contrast (**con**), excess energy in mid-range spatial frequencies (**mid-SF** or **amp**), and orientation anisotropy (**ori**), as detailed in “Methods”.

Of particular interest is the over-representation of mid-range spatial frequencies relative to the $1/f$ (orientation-averaged) amplitude spectrum characteristic of most natural images^35,36,41^. A large body of research suggests that excessive mid-SF energy may induce visual discomfort^1,32,37,38^. This has been proposed as a possible explanation for trypophobic reactions^1,24^. Because our survey was taken online, we did not explicitly control for viewing distance, but making reasonable assumptions about display sizes and viewing distances we can assume that the 256 × 256 images subtended, on average, approximately 4 degrees of visual angle. Assuming this viewing distance, we measured excess mid-SF energy in three ranges: 2 to 9 cpd (**2to9**, 8–36 cycles/image), 3 cpd +/− 1 octave (**1oct3**, 6–24 cycles/image), and 3 cpd +/− 2 octave (**2oct3**, 3–48 cycles/image), which correspond well to mid-SF definitions used in previous work^1,24,25,37^. Since contrast-reversal does not affect spatial frequency content, the measurements obtained from the Brodatz images are identical for both the pilot and main images.

**Figure 8:**
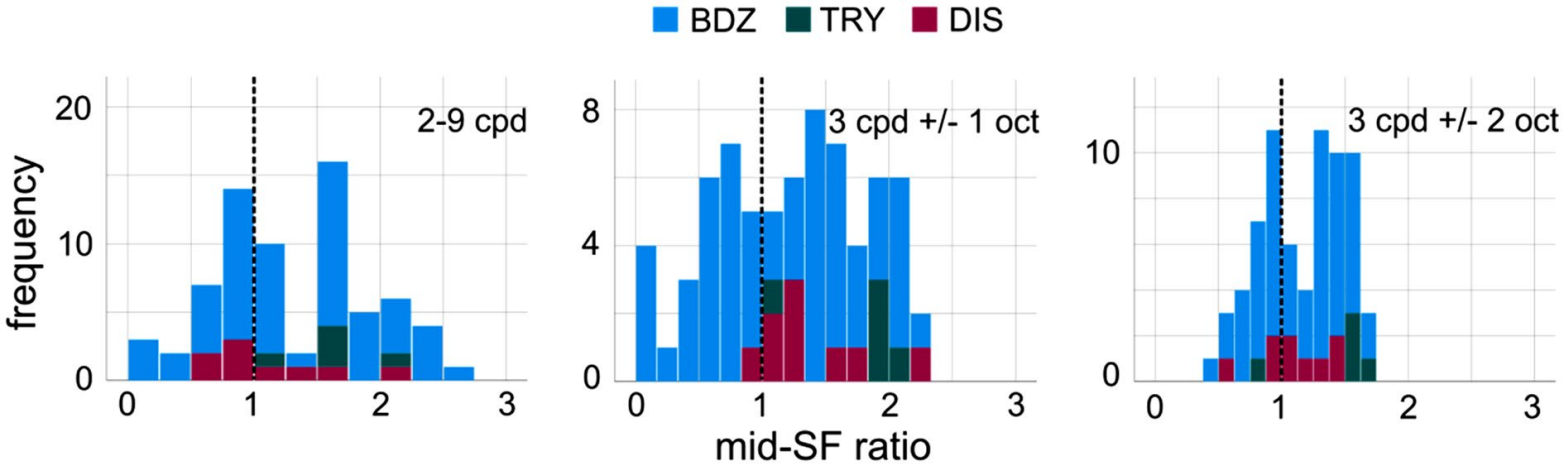
Histograms of the ratios of mid-range spatial frequency energy relative to the 1/*f* amplitude spectrum for all images. Different panels indicate 3 definitions of mid-range spatial frequencies.

Figure 8 plots the distribution of excess mid-SF energy for all three measures and for all three image categories. We find that for all image categories and all definitions of mid-SF range, the mean mid-SF ratio is greater than 1. Descriptive statistics are given in Table 1.

**Table 1:** **Mid-SF** ratios for three different definitions measured from all image categories.

	2to9		1oct3		2oct3	
	Median	Mean +/− std	Median	Mean +/− std	Median	Mean +/− std
BDZ	1.23	1.33 +/− 0.61	1.26	1.17 +/− 0.62	1.18	1.14 +/− 0.34
TRY	1.59	1.62 +/− 0.41	1.88	1.74 +/− 0.42	1.54	1.42 +/− 0.37
DIS	0.85	1.11 +/− 0.45	1.27	1.40 +/− 0.44	1.04	1.10 +/− 0.29

The mean was found to be significantly different than 1 for the N = 56 Brodatz textures for all three definitions of mid-SF ranges using the single-sample t-test (**2to9**: *t*(55) = 3.972, *p* < 0.001; **1oct3**: *t*(55) = 2.051, *p* = 0.045; **2oct3**: *t*(55) = 3.043, *p* = 0.004). For the N = 5 **TRY** textures, conventional statistical significance was obtained for 2 of the 3 definitions (t-test: **2to9**: *t*(4) = 3.39, *p* = 0.027, **1oct3**: *t*(4) = 3.916, *p* = 0.017, **2oct3**: *t*(4) = 2.525, *p* = 0.065). Finally, for the N = 9 **DIS** textures conventional significance was obtained for 1 of the 3 definitions (t-test: **2to9**: *t*(8) = 0.732, *p* = 0.485, **1oct3**: *t*(8) = 2.707, *p* = 0.027, **2oct3**: *t*(8) = 1.071, *p* = 0.316). Therefore, we see that over-representation of mid-range spatial frequency energy relative to $1/f$ is not unique to trypophobic images and seems to be a general property of natural textures more broadly.

Given the strong agreement between the three different definitions of mid-range spatial frequencies, and their tight correlations (**1oct3**-**2oct3**: *r* = 0.894, *p* < 0.001; **2to9-1oct3**: *r* = 0.879, *p* < 0.001; **2to9-2oct3**: *r* = 0.788, *p* < 0.001), we heretofore restrict our analyses to the 3 cpd +/− 1 oct definition (**1oct3**) originally proposed by Wilkins and colleagues^1,37^.

Performing a one-way ANOVA using the pilot study images yielded no differences between the mean values obtained from the three image categories (**BDZ**, **TRY**, **DIS**) for luminance (**lum**: *F*_2,67_ = 2.427, *p* = 0.096), orientation anisotropy (**ori**: *F*_2,67_ = 1.522, *p* = 0.226), or for mid-SF energy (**amp**: *F*_2,67_ = 2.555, *p* = 0.085). The only statistical measure which differed significantly between categories was RMS contrast (**con**: *F*_2,67_ = 3.383, *p* = 0.04), with the only significant pair-wise comparison being that between **BDZ** and **DIS** images (*p* = 0.035). Repeating this analysis with the main study images (where the **BDZ** textures had opposite contrast polarity) yielded similar results. No difference was observed for **lum** (*F*_2,67_ = 0.460, *p* = 0.633), **ori** (same as pilot) or **amp** (same as pilot) or RMS contrast **con** (*F*_2,67_ = 1.316, *p* = 0.275). Therefore, we see that despite the vastly different comfort ratings for these image categories (Fig. 2b), they are not well differentiated with statistics which have been previously suggested to affect visual comfort.

#### Image statistics predicting visual comfort

We next examined the variables **lum**, **con**, **ori** and **amp** for relationships with visual comfort (all participants) using a mixed effects linear model. This model was identical to that in Eq. (1), but this time the covariate was the image statistic of interest rather than TQ score. We sought to determine the relationships between each of these variables and visual comfort in both the pilot and main studies.

There was no significant main effect of **lum** on visual comfort in the pilot study (*F*_1,64_ = 1.39, *p* = 0.243), or in the main study (*F*_1,64_ = 2.075, *p* = 0.155). In the pilot study, we observed a significant effect of **lum** for the **BDZ** images (slope = − 0.0101, *t*(64) = − 4.4408, *p* < 0.001), but when these images were contrast-reversed in the main study (changing the mean luminance of the images), a significant effect observed in the opposite direction (slope = 0.01159, *t*(64) = 3.696, *p* < 0.001). A very similar finding was obtained for analysis of contrast (**con**), where we failed to observe any significant main effects in the pilot (*F*_1,64_ = 0.281, *p* = 0.598) and main (*F*_1,64_ = 2.196, *p* = 0.143), but observed significant effects for the **BDZ** textures in the pilot (slope = 1.239, *t*(64) = 2.239, *p* = 0.029) and main (slope = -1.798, *t*(64) = − 2.920, *p* = 0.005) in opposite directions. Given that **con** and **lum** exhibited strong negative correlations in the **BDZ** database, the most likely explanation for our results is that the **BDZ** images eliciting the greatest visual discomfort in our pilot study (for reasons unrelated to their luminance or their RMS contrast) may have happened to have lower luminance (and higher contrast). Therefore, when they were contrast-reversed, we would expect the lower contrast images to elicit more discomfort, leading to an effect in the opposite direction.

We next considered **amp** and **ori** statistics, which have been previously implicated in the literature on visual discomfort^29,30,37^. In the pilot study, we did not observe any overall significant effect of **amp** on viewing comfort (*F*_1,64_ = 0.113, *p* = 0.738), and did not observe significant effects for any category (**BDZ**: *t*(64) = − 0.452, *p* = 0.653; **TRY**: *t*(64) = − 0.493, *p* = 0.624; **DIS**: *t*(64) = 1.455, *p* = 0.151). Similarly, in the main study there was no effect of **amp** on viewing comfort, either overall (*F*_1,64_ = 0.0666,* p* = 0.797) or for any image category (**BDZ**: *t*(64) = 1.675, *p* = 0.099; **TRY**: *t*(64) = − 0.551, *p* = 0.584; **DIS**: *t*(64) = 0.819, *p* = 0.416). In the pilot study, we also did not find any significant effect of **ori**, either overall (F_1,64_ = 0.00337, *p* = 0.954) or for any subset of images (**BDZ**: *t*(64) = 1.7746,* p* = 0.081; **TRY**: *t* = 0.0937, *p* = 0.926; **DIS**: *t* = − 0.7507, *p* = 0.456). However in the main study, which had greater statistical power than the pilot, although there was no overall significance (*F*_1,64_ = 0.185, *p* = 0.669), we did observe for the **BDZ** textures a slight positive trend which reached statistical significance (*t*(64) = 2.434, *p* = 0.018). This is consistent with previous observations showing increased orientation anisotropy to be positively associated with viewing comfort^29,30^.

The general inability of the **amp** and **ori** statistics to explain visual comfort in a wide variety of natural textures images is very much consistent with previous work showing vastly different visual comfort ratings for trypophobic textures and phase-scrambled noise (**PSC**) generated from these textures^25^. By construction, each of these images and its phase-scrambled counterpart has an identical amplitude spectrum, and therefore identical orientation and spatial frequency content. However, we found that the **PSC** textures were significantly more comfortable to view than the **TRY** textures^25^. These previous results, taken with our current analysis, argue strongly against low-level image statistics as being the primary determinant of visual discomfort elicited by trypophobic imagery (and textures more generally), and support more complex texture-recognition processes, possibly mediated by dedicated texture-processing visual brain areas^42^.

## Discussion

### Trypophobia, visual texture and disease

Since its initial description in scientific literature, several theories have been proposed as explanations for trypophobic visual discomfort. Cole and Wilkins^1^ originally suggested that the visual discomfort elicited by trypophobic imagery arises from its over-representation of mid-range spatial frequencies, which they demonstrated are also over-represented in images of many dangerous animals, such as snakes and spiders. In their view, the aversion to mid-range spatial frequencies like those commonly observed in trypophobic imagery represents an evolved adaptation to help organisms avoid dangerous animals. However, another framework has received more recent support, namely that trypophobia represents an over-generalized disease avoidance response, as trypophobic stimuli (Fig. 1a,b) visually resemble various skin disease images (Fig. 1c) which often contain clusters of irregular circular bumps or holes^3^. This hypothesis has also been referred to as the “involuntary protection against dermatosis” hypothesis^13^ or “skin-disease avoidance” hypothesis^6^. Consistent with this idea, several studies have shown that the aversive responses to trypophobic imagery are best characterized as a disgust response^3,43,44^, an emotion which most likely evolved to serve a functional role of avoiding disease and contamination^11^. Furthermore, other work has shown greater aversion to trypophobic imagery in those individuals who had previously experienced skin disease^13^, and that priming with skin-disease relevant words increases the visual discomfort elicited by trypophobic imagery^8^.

In the current study, we reproduced the general findings of Kupfer & Le^3^, demonstrating that while all individuals find disease images aversive (and lower in comfort compared to **BDZ** images), only those scoring high on the **TQ** find the trypophobic images to be equally aversive as disease images. Comparable results were seen in both samples studied and analyzing the data using traditional ANOVA models as well as with linear mixed effects modeling. Interestingly, our mixed effects model analyses (Fig. 3) revealed that there is a statistically significant effect of TQ score on viewing comfort for images of disease. This suggests that individuals with trypophobia may be more sensitive to disease images than other individuals, which is consistent with the idea of trypophobia being a generalized disease avoidance response^3^. Finally, it is noteworthy that even people who score low on the TQ find trypophobic images less pleasant than texture images more generally, a phenomenon which has been reported in several previous studies^3–5,25^.

### Texture aesthetics and trypophobia

The current study further extends the work of Kupfer and Le^3^ by including a large and robust set of standard visual textures^40^, used in previous research as comparison or control stimuli. Although some investigators have used a limited set of textures as comparison stimuli^3^, most studies, including our own work, have presented non-textured images containing holes as control stimuli, for instance a picture of a golf ball hole or cannon barrel^1,2,6,25^. Since both trypophobic and skin disease images are examples of natural textures, we suggest that the most appropriate comparison stimuli for characterizing trypophobic visual discomfort would be a representative set of natural textures, which we approximate with a set of Brodatz textures from classic work on the perceptual dimensions of texture^34^. To our knowledge, the present study is the first to situate studies of trypophobia in the more general context of texture aesthetics, a broad field with implications not only for academic neuroscience but also more applied areas like art, fashion, and design^22^.

We find that all populations of observers find that on average, the texture stimuli are similarly comfortable to view (Fig. 2b–d, *white circles*), at least when compared to the disease and trypophobic images, in which case both groups find highly uncomfortable to view. Even though the textures are rated as being neutral or slightly positive on average, there was significant variation between textures (Fig. 4). Although the exact quantitative image features responsible for these comfort ratings are unknown, we may note that the most pleasant textures were low-density images with clearly visible objects like the floral prints, as well as highly regular, low-density, repetitive directional textures like the brick walls (Fig. 5). Similarly, for all observers the least pleasant textures tended to be high-density, irregular textures with circular bumps or holes (Fig. 5). Furthermore, we find that both **hi-TQ** and **lo-TQ** populations rate the visual comfort of textures similarly (Fig. 6). In fact, only a handful of the Brodatz textures significantly distinguish these two populations (Fig. 7), and as one might expect they are those textures which are comprised of irregular clusters of bumps, much like the disease and trypophobic images (Fig. 1). This finding is consistent with the idea that trypophobia is a generalized disease avoidance response. Although a complete characterization of texture aesthetics is beyond the scope of the present work, the obvious implication of our results for artists and designers is that irregular, high-density patterns with clusters of holes are likely to be at least somewhat uncomfortable for most viewers and may even run the risk of inducing trypophobic responses in a sizeable minority of the population.

### Image statistics and visual discomfort

Natural images occupy a tiny fraction of the vast space of theoretically possible images^45^. Therefore, a working hypothesis which has motivated sensory neuroscience for many decades is the idea that the visual system is adapted to efficiently represent this space of natural images^45,46^. Since neural activity is metabolically demanding^47^, efficient coding theory posits that the goal of the nervous system is to represent visual information accurately using the smallest number of active neurons, a strategy known as *sparse coding*^48,49^. One robust feature of natural images is that they are scale-invariant, meaning that they have equal energy in different spatial frequency bands, which implies that their orientation-averaged amplitude spectrum has a $1/f$ shape, or equivalently has a -1 slope when plotted in log–log coordinates^35^. If the visual system has adapted to represent such images in an efficient manner, then it follows that images which deviate from these statistics will not be represented efficiently, that is, they will require many active neurons.

Researchers studying visual discomfort have proposed that images which are uncomfortable to view are precisely those which over-activate the visual cortex, a hypothesis referred to as the cortical hypermetabolism hypothesis^23,32,37,38,50^. Since the visual cortex is maximally sensitive to mid-range spatial frequencies^51,52^, one prediction of this hypermetabolism hypothesis is that stimuli which over-represents such frequencies relative to their predicted levels in natural imagery ($1/f$ amplitude spectrum) would cause excessive cortical activity and lead to visual discomfort. Several previous investigations have lent support to this notion, including those with striped patterns^38^, artworks^37^, man-made structures^50^, noise textures^28,29^, and a wide assortment of photographic images^32^. In addition to these experiments, simulations of populations of V1 neurons having similar tuning properties to those found in vivo exhibited greater activity when presented with images over-representing mid-range spatial frequencies^53^, consistent with the hypermetabolism hypothesis.

Since trypophobic images over-represent mid-range spatial frequencies relative to $1/f$, one proposed basis for trypophobic visual discomfort has been this over-representation of such frequencies^1,23,24^. However, more recent research into trypophobia suggests that the hypermetabolism hypothesis might be short-sighted as a full explanation for trypophobic visual discomfort. In a direct test of this hypothesis, Pipitone and DiMattina^25^ compared the visual comfort ratings of trypophobic imagery with those of phase-scrambled versions of those same images. Whereas observers found the original trypophobic images as well as the original phase / 1/*f* amplitude images rather uncomfortable to view, they did not find the phase-scrambled versions as uncomfortable to view. Since both the original and phase-scrambled versions have identical amplitude spectrum content, this argues against the low-level amplitude spectrum information as being the primary cause of trypophobic reactions. Consistent with these findings, another study found that while trypophobic imagery captured attention in a binocular masking task, phase-scrambled versions of these same images did not, despite having identical amplitude spectra^26^. Finally, an earlier study by Le et al.^2^ which defined the TQ found that there was little change in the visual discomfort induced by trypophobic imagery when the amplitude spectrum was changed to $1/f$. These findings argue against the amplitude spectrum characteristics as being the primary determinants of visual discomfort in trypophobia.

In the present study, we show additional evidence that low-level image statistics which are captured in the Fourier amplitude spectrum are not the primary determinant of visual comfort for natural textures more generally. Using three definitions of mid-range spatial frequencies, we failed to observe a significant correlation between the over-representation of these frequencies and viewing comfort. Furthermore, we see that comfort ratings for **TRY** and **DIS** stimuli are qualitatively different in general, being rated as much less comfortable than **BDZ** textures which have similar degrees of over-representation. This does not invalidate the cortical hypermetabolism hypothesis for images but does argue against it as a mechanistic explanation for the discomfort elicited by these texture image categories (**BDZ**, **TRY**, **DIS**). Furthermore, comparing the amplitude spectrum measured from trypophobic imagery and the other image categories does not reveal a significant difference in excess mid-SF energy with image categories (Fig. 8). That is, textures generally over-represent mid-range SFs, whereas only a very limited subset of textures evoke trypophobia responses. This argues further against this over-representation of mid-SF energy as the primary determinant of trypophobic reactions.

Another interesting observation which speaks to the larger literature on visual discomfort is that we do not find a strong association between orientation anisotropy and visual comfort reported in a recent study^29^. We define a novel measure of orientation anisotropy for natural images and measured it from our image sets. We were only able to obtain a statistically significant effect for the **BDZ** textures in the main study, which was in the same direction as previously reported^29,30^.

The lack of predictive power of low-level image statistics readily measurable from the amplitude spectrum of our textures is somewhat surprising given that previous work has demonstrated that deviations of natural images from a $1/f$ amplitude spectrum can account for at least some of the variability in comfort ratings of natural images. However, it is very much consistent with our previous work of trypophobic imagery, which revealed that two images with identical amplitude spectra can have vastly different comfort ratings^25^. This held for both trypophobic imagery, where the phase-scrambled controls were significantly more comfortable to view, as well as control images, whereas the amplitude-scrambled / original phase images were significantly less comfortable to view, comparable to the original images.

### Different processes may mediate different kinds of discomfort

There are many ways an image can potentially be discomforting. The visual discomfort associated with cortical hypermetabolism is best described as having excessive glare or invoking a headache^38^, and hypermetabolism may be caused by a wide variety of images, including stripes, noise patterns, artwork, and various natural scenes. By contrast, the primary feeling associated with trypophobia seems to be disgust^3,7,54^, and trypophobic responses are specific to a subset of visual textures containing clusters of circular objects/holes. Therefore, it is plausible that these different forms of visual discomfort may have a different mechanistic basis in the brain.

It is known that there are brain areas exhibiting some degree of specialization to texture stimuli^39,42,55,56^. Since texture provides potentially important information about material^20^, and some materials may be harmful (for instance, diseased skin), it is not implausible that complex texture recognition processes may be the major determinant of visual comfort ratings for textured stimuli, perhaps combining with lower-level phenomena and involves cortical hypermetabolism (see Ref.^23^). Indeed, recent work on the perception of material properties has demonstrated that observers can reliably perceive the moisture context of a material and can exhibit feelings of disgust or aversion to a range of specific moistures^57^. By contrast, for more general sets of images without dedicated processing mechanisms for evaluating potential harm, the main determinant of visual comfort may be the degree of cortical hyper-metabolism in lower-level visual areas (V1) evoked by the stimulus. This idea would neatly reconcile the results in our study with previous work using natural images and would elegantly explain the vast under-prediction of the visual discomfort elicited by **TRY** and **DIS** images in this study.

In summary, we propose the hypothesis that visual mechanisms exist for the detection of skin disease textures which provide inputs into various brain regions responsible for evoking a disgust response. It would be of great interest for future neuroimaging studies to identify brain regions which exhibit differential sensitivity to disease-relevant textures and disease-irrelevant texture stimuli. If such regions could be identified, one might expect that trypophobic individuals will have similar biological and subjective responses not only to actual disease images but also by images exhibiting some degree of visual similarities to disease. We hope that the present work serves to motivate research to test this hypothesis.

### Supplementary Information

Supplementary Tables.

## Data Availability

The datasets used and/or analyzed during the current study are available from the corresponding author on reasonable request.

## References

[1] Cole GG, Wilkins AJ (2013). Fear of holes. Psychol. Sci..

[2] Le ATD, Cole GG, Wilkins AJ (2015). Assessment of trypophobia and an analysis of its visual precipitation. Q. J. Exp. Psychol..

[3] Kupfer TR, Le ATD (2018). Disgusting clusters: Trypophobia as an overgeneralised disease avoidance response. Cognit. Emot..

[4] Can W, Zhuoran Z, Zheng J (2017). Is trypophobia a phobia?. Psychol. Rep..

[5] Pipitone RN, Gallegos B, Walters D (2017). Physiological responses to trypophobic images and further scale validity of the trypophobia questionnaire. Personal. Individ. Differ..

[6] Pipitone RN, DiMattina C, Martin ER, Pavela Banai I, Bellmore KL, de Angelis M (2022). Evaluating the ‘skin disease-avoidance’ and ‘dangerous animal’ frameworks for understanding trypophobia. Cognit. Emot..

[7] Furuno M, Imaizumi S, Maeda K, Hibono H, Koyama S (2017). The influence of background objects on unpleasantness evoked by lotus-seed-pods-on-the-living-body Images (&ldquo;Hasu-colla&rdquo;). Int. J. Affect. Eng..

[8] Shirai R, Ogawa H (2021). Priming with skin-problems increases fear of clusters. Sci. Rep..

[9] Kupfer TR, Fessler DMT, Wu B, Hwang T, Sparks AM, Alas S, Samore T, Lal V, Sakhamuru TP, Holbrook C (2021). The skin crawls, the stomach turns: Ectoparasites and pathogens elicit distinct defensive responses in humans. Proc. R. Soc. B.

[10] Kupfer TR, Fessler DM (2018). Ectoparasite defense in humans: Relationships to pathogen avoidance and clinical implications. Philos. Trans. R. Soc. B Biol. Sci..

[11] Oaten M, Stevenson RJ, Case TI (2009). Disgust as a disease-avoidance mechanism. Psychol. Bull..

[12] Schaller M, Park JH (2011). The behavioral immune system (and why it matters). Curr. Dir. Psychol. Sci..

[13] Yamada Y, Sasaki K (2017). Involuntary protection against dermatosis: A preliminary observation on trypophobia. BMC Res. Notes.

[14] Bergen, J. R. Theories of visual texture perception. *Vis. Visual Dysfunct. 10B Spatial Vis.* 0(0), 114–134 (1991).

[15] DiMattina C, Baker CL (2019). Modeling second-order boundary perception: A machine learning approach. PLoS Comput. Biol..

[16] Victor JD, Conte MM, Chubb CF (2017). Textures as probes of visual processing. Annu. Rev. Vis. Sci..

[17] Zavitz E, Baker CL (2013). Texture sparseness, but not local phase structure, impairs second-order segmentation. Vis. Res..

[18] Adelson EH, Rogowitz BE, Pappas TN (2001). On seeing stuff: The perception of materials by humans and machines. Human Vision and Electronic Imaging VI.

[19] Adelson EH, Bergen JR (1991). The Plenoptic Function and the Elements of Early Vision.

[20] Motoyoshi I, Nishida S, Sharan L, Adelson EH (2007). Image statistics and the perception of surface qualities. Nature.

[21] Portilla J, Simoncelli EP (2000). A parametric texture model based on joint statistics of complex wavelet coefficients. Int. J. Comput. Vis..

[22] Jacobs RHAH, Haak KV, Thumfart S, Renken R, Henson B, Cornelissen FW (2016). Aesthetics by numbers: Links between perceived texture qualities and computed visual texture properties. Front. Hum. Neurosci..

[23] Le A, Cole GG, Wilkins A (2020). Trypophobia: Heart rate, heart rate variability and cortical haemodynamic response. J. Affect. Disord..

[24] Sasaki K, Yamada Y, Kuroki D, Miura K (2017). Trypophobic discomfort is spatial-frequency dependent. Adv. Cognit. Psychol..

[25] Pipitone RN, DiMattina C (2020). Object clusters or spectral energy? Assessing the relative contributions of image phase and amplitude spectra to trypophobia. Front. Psychol..

[26] Shirai R, Ogawa H (2019). Trypophobic images gain preferential access to early visual processes. Conscious. Cognit..

[27] Isherwood ZJ, Schira MM, Spehar B (2017). The tuning of human visual cortex to variations in the 1/fα amplitude spectra and fractal properties of synthetic noise images. NeuroImage.

[28] Juricevic I, Land L, Wilkins A, Webster MA (2010). Visual discomfort and natural image statistics. Perception.

[29] Ogawa N, Motoyoshi I (2020). Differential effects of orientation and spatial-frequency spectra on visual unpleasantness. Front. Psychol..

[30] Ogawa N, Motoyoshi I (2021). Spatiotemporal frequency characteristics of the visual unpleasantness of dynamic bandpass noise. Vis. Res..

[31] O’Hare L, Hibbard PB (2011). Spatial frequency and visual discomfort. Vis. Res..

[32] Penacchio O, Wilkins AJ (2015). Visual discomfort and the spatial distribution of Fourier energy. Vis. Res..

[33] Rao AR, Lohse GL (1993). Towards a texture naming system: Identifying relevant dimensions of texture. Proc. 4th Conf. Vis..

[34] Rao AR, Lohse GL (1996). Towards a texture naming system: Identifying relevant dimensions of texture. Vis. Res..

[35] Field DJ (1987). Relations between the statistics of natural images and the response properties of cortical cells. J. Opt. Soc. Am. A.

[36] van der Schaaf A, van Hateren JH (1996). Modelling the power spectra of natural images: Statistics and information. Vis. Res..

[37] Fernandez D, Wilkins AJ (2008). Uncomfortable images in art and nature. Perception.

[38] Wilkins A, Nimmo-smith I, Tait A, Mcmanus C, Sala SD, Tilley A, Arnold K, Barrie M, Scott S (1984). A neurological basis for visual discomfort. Brain.

[39] Jacobs RHAH, Baumgartner E, Gegenfurtner KR (2014). The representation of material categories in the brain. Front. Psychol..

[40] Brodatz P (1966). Textures: A Photographic Album for Artists and Designers.

[41] Ruderman DL (1997). Origins of scaling in natural images. Vis. Res..

[42] Cant JS, Goodale MA (2007). Attention to form or surface properties modulates different regions of human occipitotemporal cortex. Cereb. Cortex.

[43] Furuno M, Sakurai Y, Imaizumi S, Koyama S (2018). Face-inversion effect on disgust evoked by a cluster of dots. I-Perception.

[44] Imaizumi S, Furuno M, Hibino H, Koyama S (2016). Trypophobia is predicted by disgust sensitivity, empathic traits, and visual discomfort. Springerplus.

[45] Field DJ (1994). What is the goal of sensory coding?. Neural Comput..

[46] Barlow H (2001). Redundancy reduction revisited. Netw. Comput. Neural Syst..

[47] Lennie P (2003). The cost of cortical computation. Curr. Biol..

[48] Olshausen BA, Field DJ (2004). Sparse coding of sensory inputs. Curr. Opin. Neurobiol..

[49] Olshausen BA, Fieldt DJ (1997). Sparse coding with an overcomplete basis set: A strategy employed by V1? Coding V1 gabor-wavelet natural images. Vis. Res..

[50] Le ATD, Payne J, Clarke C, Kelly MA, Prudenziati F, Armsby E, Penacchio O, Wilkins AJ (2017). Discomfort from urban scenes: Metabolic consequences. Landsc. Urban Plann..

[51] Foster KH, Gaska JP, Nagler M, Pollen DA (1985). Spatial and temporal frequency selectivity of neurones in visual cortical areas V1 and V2 of the macaque monkey. J. Physiol..

[52] Parker AJ, Hawken MJ (1988). Two-dimensional spatial structure of receptive fields in monkey striate cortex. J. Opt. Soc. Am. A.

[53] Hibbard PB, O’Hare L (2015). Uncomfortable images produce non-sparse responses in a model of primary visual cortex. R. Soc. Open Sci..

[54] Mayor E, Meyer A, Miani A, Lieb R (2021). An exploration of the nomological network of trypophobia. PLOS ONE.

[55] Cavina-Pratesi C, Kentridge RW, Heywood CA, Milner AD (2010). Separate channels for processing form, texture, and color: Evidence from fMRI adaptation and visual object agnosia. Cereb. Cortex.

[56] Goda N, Tachibana A, Okazawa G, Komatsu H (2014). Representation of the material properties of objects in the visual cortex of nonhuman primates. J. Neurosci..

[57] Iwasa K, Komatsu T, Kitamura A, Sakamoto Y (2020). Visual perception of moisture is a pathogen detection mechanism of the behavioral immune system. Front. Psychol..

