# Supplementary material for: Trypophobia, skin disease, and the visual discomfort of natural textures
Source: Sci Rep. 2024 Feb 29;14:5050. doi: 10.1038/s41598-024-55149-8 (PMC10904841; doi:10.1038/s41598-024-55149-8)
Supplement: Supplementary file 1 — Supplementary Tables. [file 41598_2024_55149_MOESM1_ESM.docx]

**Supplementary Table 1**

| **Post Hoc Comparisons - group ✻ Image Type** | | | | | | | | | | | |
| --- | --- | --- | --- | --- | --- | --- | --- | --- | --- | --- | --- |
|  | |  | | **Mean Difference** | | **SE** | | **t** | | **p _holm_** | |
| 1, BDZ |  | 2, BDZ |  | 0.527 |  | 0.184 |  | 2.870 |  | 0.009 |  |
|  |  | 1, TRY |  | 2.531 |  | 0.125 |  | 20.279 |  | < .001 |  |
|  |  | 2, TRY |  | 4.662 |  | 0.184 |  | 25.380 |  | < .001 |  |
|  |  | 1, DIS |  | 3.457 |  | 0.125 |  | 27.699 |  | < .001 |  |
|  |  | 2, DIS |  | 4.531 |  | 0.184 |  | 24.665 |  | < .001 |  |
| 2, BDZ |  | 1, TRY |  | 2.004 |  | 0.184 |  | 10.909 |  | < .001 |  |
|  |  | 2, TRY |  | 4.135 |  | 0.125 |  | 33.128 |  | < .001 |  |
|  |  | 1, DIS |  | 2.930 |  | 0.184 |  | 15.951 |  | < .001 |  |
|  |  | 2, DIS |  | 4.004 |  | 0.125 |  | 32.076 |  | < .001 |  |
| 1, TRY |  | 2, TRY |  | 2.131 |  | 0.184 |  | 11.601 |  | < .001 |  |
|  |  | 1, DIS |  | 0.926 |  | 0.125 |  | 7.420 |  | < .001 |  |
|  |  | 2, DIS |  | 2.000 |  | 0.184 |  | 10.886 |  | < .001 |  |
| 2, TRY |  | 1, DIS |  | -1.205 |  | 0.184 |  | -6.559 |  | < .001 |  |
|  |  | 2, DIS |  | -0.131 |  | 0.125 |  | -1.052 |  | 0.293 |  |
| 1, DIS |  | 2, DIS |  | 1.074 |  | 0.184 |  | 5.844 |  | < .001 |  |
|  | | | | | | | | | | | |
| *Note.*  P-value adjusted for comparing a family of 15 | | | | | | | | | | | |

**Supplementary Table 2**

| **Post Hoc Comparisons - group ✻ Image Type** | | | | | | | | | | | |
| --- | --- | --- | --- | --- | --- | --- | --- | --- | --- | --- | --- |
|  | |  | | **Mean Difference** | | **SE** | | **t** | | **p _holm_** | |
| 1, BDZ |  | 2, BDZ |  | 0.633 |  | 0.295 |  | 2.143 |  | 0.033 |  |
|  |  | 1, TRY |  | 2.079 |  | 0.175 |  | 11.848 |  | < .001 |  |
|  |  | 2, TRY |  | 5.401 |  | 0.295 |  | 18.280 |  | < .001 |  |
|  |  | 1, DIS |  | 3.179 |  | 0.175 |  | 18.119 |  | < .001 |  |
|  |  | 2, DIS |  | 4.922 |  | 0.295 |  | 16.658 |  | < .001 |  |
| 2, BDZ |  | 1, TRY |  | 1.445 |  | 0.295 |  | 4.892 |  | < .001 |  |
|  |  | 2, TRY |  | 4.768 |  | 0.175 |  | 27.177 |  | < .001 |  |
|  |  | 1, DIS |  | 2.546 |  | 0.295 |  | 8.616 |  | < .001 |  |
|  |  | 2, DIS |  | 4.289 |  | 0.175 |  | 24.445 |  | < .001 |  |
| 1, TRY |  | 2, TRY |  | 3.322 |  | 0.295 |  | 11.245 |  | < .001 |  |
|  |  | 1, DIS |  | 1.100 |  | 0.175 |  | 6.271 |  | < .001 |  |
|  |  | 2, DIS |  | 2.843 |  | 0.295 |  | 9.623 |  | < .001 |  |
| 2, TRY |  | 1, DIS |  | -2.222 |  | 0.295 |  | -7.521 |  | < .001 |  |
|  |  | 2, DIS |  | -0.479 |  | 0.175 |  | -2.732 |  | 0.013 |  |
| 1, DIS |  | 2, DIS |  | 1.743 |  | 0.295 |  | 5.899 |  | < .001 |  |
|  | | | | | | | | | | | |
| *Note.*  P-value adjusted for comparing a family of 15 | | | | | | | | | | | |

**Supplementary Table 3**

**PILOT**

| Fixed Effect Omnibus tests | | | | | | | | | |
| --- | --- | --- | --- | --- | --- | --- | --- | --- | --- |
|  | | **F** | | **Num df** | | **Den df** | | **p** | |
| Image_Type |  | 93.00 |  | 2 |  | 67.0 |  | < .001 |  |
| TQ_score |  | 9.76 |  | 1 |  | 93.6 |  | 0.002 |  |
| Image_Type ✻ TQ_score |  | 58.29 |  | 2 |  | 6135.0 |  | < .001 |  |
| Note. Satterthwaite method for degrees of freedom | | | | | | | | | |
|  | | | | | | | | | |

| Random Components | | | | | | | | | |
| --- | --- | --- | --- | --- | --- | --- | --- | --- | --- |
| **Groups** | | **Name** | | **SD** | | **Variance** | | **ICC** | |
| Subject_ID |  | (Intercept) |  | 1.376 |  | 1.894 |  | 0.408 |  |
| Item_ID |  | (Intercept) |  | 0.806 |  | 0.650 |  | 0.192 |  |
| Residual |  |  |  | 1.656 |  | 2.743 |  |  |  |
| Note. Number of Obs: 6296 , groups: Subject_ID 90, Item_ID 70 | | | | | | | | | |
|  | | | | | | | | | |

**MAIN**

| Fixed Effect Omnibus tests | | | | | | | | | |
| --- | --- | --- | --- | --- | --- | --- | --- | --- | --- |
|  | | **F** | | **Num df** | | **Den df** | | **p** | |
| TQ_score |  | 42.1 |  | 1 |  | 213.3 |  | < .001 |  |
| Image_Type |  | 66.8 |  | 2 |  | 67.0 |  | < .001 |  |
| TQ_score ✻ Image_Type |  | 121.9 |  | 2 |  | 13514.0 |  | < .001 |  |
| Note. Satterthwaite method for degrees of freedom | | | | | | | | | |
|  | | | | | | | | | |

| Random Components | | | | | | | | | |
| --- | --- | --- | --- | --- | --- | --- | --- | --- | --- |
| **Groups** | | **Name** | | **SD** | | **Variance** | | **ICC** | |
| Subject_ID |  | (Intercept) |  | 1.19 |  | 1.41 |  | 0.320 |  |
| Item_ID |  | (Intercept) |  | 1.07 |  | 1.14 |  | 0.275 |  |
| Residual |  |  |  | 1.73 |  | 3.01 |  |  |  |
| Note. Number of Obs: 13782 , groups: Subject_ID 197, Item_ID 70 | | | | | | | | | |
|  | | | | | | | | | |

**POOLED**

| Fixed Effect Omnibus tests | | | | | | | | | |
| --- | --- | --- | --- | --- | --- | --- | --- | --- | --- |
|  | | **F** | | **Num df** | | **Den df** | | **p** | |
| Image_Type |  | 82.7 |  | 2 |  | 67.0 |  | < .001 |  |
| TQ_score |  | 54.4 |  | 1 |  | 306.4 |  | < .001 |  |
| Image_Type ✻ TQ_score |  | 169.9 |  | 2 |  | 19513.0 |  | < .001 |  |
| Note. Satterthwaite method for degrees of freedom | | | | | | | | | |
|  | | | | | | | | | |

| Random Components | | | | | | | | | |
| --- | --- | --- | --- | --- | --- | --- | --- | --- | --- |
| **Groups** | | **Name** | | **SD** | | **Variance** | | **ICC** | |
| Subject_ID |  | (Intercept) |  | 1.246 |  | 1.553 |  | 0.338 |  |
| Item_ID |  | (Intercept) |  | 0.929 |  | 0.864 |  | 0.221 |  |
| Residual |  |  |  | 1.743 |  | 3.036 |  |  |  |
| Note. Number of Obs: 19868 , groups: Subject_ID 284, Item_ID 70 | | | | | | | | | |
|  | | | | | | | | | |
